# Comparative Study of Various Delivery Methods for the Supply of Alpha-Ketoglutarate to the Neural Cells for Tissue Engineering

**DOI:** 10.1155/2013/294679

**Published:** 2013-06-26

**Authors:** Tanushree Vishnoi, Ashok Kumar

**Affiliations:** ^1^Department of Biological Sciences and Bioengineering, Indian Institute of Technology Kanpur, Kanpur 208016, India; ^2^Thematic Unit of Excellence on Nanoscience and Nanotechnology, Indian Institute of Technology Kanpur, Kanpur 208016, India

## Abstract

Delivery of growth factors or bioactive molecules plays an important role in tissue engineering, as the duration to which these are supplied can modulate the cell fate. Thus, the delivery method plays an important role, and the same is presented in this work wherein the exogenous supply of alpha-ketoglutarate (**α**-KG) gave better results for fast proliferating cells as compared to delivery by microspheres or microspheres incorporated scaffolds which can be used while culturing slow growing cells. All these studies were performed in two dimensional (2D) and three dimensional (3D) setups in which chitosan-gelatin-polypyrrole has been used as 3-D scaffolds. Chitosan and gelatin microspheres alone as well as incorporated in the cryogels were characterized. MTT assay done using neuro-2a cell line showed approximately 42% and 70% increment in cellular proliferation when gelatin and chitosan microspheres were added in a 3-D setup, respectively, as compared to the control. Biochemical analysis of ammonia showed 6-fold reductions in ammonia level in a 3-D setup compared to the control. We also studied the synthesis of a neurotransmitter-like glutamate and found that its concentration increased up to 0.25 mg/ml when the microspheres were added exogenously in a 3-D system.

## 1. Introduction

Neural cells are well known-excitable cells which undergo activation and deactivation via stimulus from the external environment. These cells upon excitation release neurotransmitters which further signal the other neural cells and thus propagate the stimulus unidirectionally. There are excitatory as well as inhibitory neurotransmitters like acetylcholine, glutamate, and so forth and *γ*-amino butyric acid (GABA), respectively [1]. It has been reported that when alpha-ketoglutarate (*α*-KG), an intermediate in the citric acid cycle (TCA cycle), is added exogenously in the media, it leads to the formation of amino acids like glutamate and glutamine [2]. Glutamine is involved in the protein synthesis, whereas glutamate is an important neurotransmitter. *α*-KG also acts as a scavenger for ammonia, thereby converting the ammonia to glutamate and then to glutamine [3]. Apart from this, *α*-KG is known to be a potent natural detoxifying agent [4] as well as have been shown to play an important role in collagen metabolism [5]. Considering the varied effect of *α*-KG, we have previously studied its effect in the production of monoclonal antibody, wherein it was found that *α*-KG not only increased the viable cell density by scavenging ammonia but it also increased the initial monoclonal antibody production [6]. Therefore in this work we tried to explore the various methods of *α*-KG delivery to the cells in order to achieve maximum delivery to the cells and thus efficient growth, proliferation and neurotransmitter production.

There has been a significant research work in the field of delivery of growth factors [7] and bioactive molecules [8] for the improvement of *in vitro* growth and proliferation of cells for regeneration during injury/disease in tissue engineering applications. These molecules enable the cells to grow and survive better either by acting as a signaling molecule or as a potential metabolite which enables the cell improved growth, proliferation, and differentiation. Although exogenous supply of molecules has long been followed, they have their own disadvantages like solubility, long term availability, and so forth [9]. Therefore, in order to overcome these disadvantages, delivery via microspheres, synthesized either by natural or synthetic polymers, has been designed. These microspheres enable the delivery of the molecule either by osmotic burst, diffusion or degradation/erosion of the polymer [10]. The polymers used for the synthesis of these microspheres can be both degradable [11] as well as nondegradable [12]. Incorporation of the molecules in these microspheres allows controlled diffusion and exposes the cells with the molecule for longer duration. In order to study the effect of different delivery methods on the cells, we employed another method wherein we incorporated the *α*-KG containing microspheres inside the three-dimensional (3-D) cryogel scaffolds, synthesized via cryogelation technology. Cryogels are macroporous gels synthesized at subzero temperature leading to properly interconnected structure which allows proper flow of nutrients and waste to and from the cells [13]. The cryogels after synthesis are thawed at room temperature which results in the melting of ice crystals, and thus provide pore formation [14]. These pores lead to a larger surface area for the microspheres to be incorporated. Moreover, the size of these pores can be regulated by controlling the polymer concentration and freezing temperature [15]. Incorporation of these microspheres in the cryogel will vary the release of the *α*-KG in the medium and thus the amount to which the cells are exposed. 

In this study, we synthesized microspheres from two kinds of natural polymers like chitosan and gelatin differing in their rate of degradation. Gelatin is known to have a higher rate of degradation compared to chitosan [16] and thus will expose the cells to different amounts of *α*-KG over the same period of time to which the cells are exposed. Therefore we tried to explore the effect of varied delivery methods like: (1) exogenous free supply; (2) delivery via microspheres in both 2-D and 3-D systems; (3) *α*-KG loaded microspheres incorporated in the cryogel to study the effect of *α*-KG on glucose and ammonia metabolism as well as the neurotransmitter production in order to achieve better results in cell culture and tissue engineering applications.

## 2. Materials

Chitosan (low viscosity), gelatin (from cold water fish skin, MW: ~60,000), Dulbecco's modified medium (DMEM), penicillin-streptomycin antibiotic, 3-(4,5-dimethylthiazol-2-yl)-2,5-diphenyl tetrazolium bromide (MTT), and fluorescein diacetate (FDA) were bought from Sigma Chemical Co. (St. Louis, MO, USA). Foetal bovine serum (FBS) was procured from HyClone (Utah, USA). Sodium dodecyl sulfate (SDS), ammonium persulfate (APS), light and heavy paraffin, isopropanol, n-hexane, methanol, acetonitrile, and toluene were procured from Merck Chemical Co. (Mumbai, India). Glutaraldehyde and *β*-mercaptoethanol were purchased from S.D.Fine Chemicals Limited (Mumbai, India). Iron chloride (FeCl_3_) was bought from Hi-Media (Mumbai, India). Pyrrole and o-pthalaldehyde were procured from Spectrochem (Andhra Pradesh, India). Span 80 was purchased from Loba Chemie (Mumbai, India). Chloroform was bought from Fisher Scientific (Mumbai, India). 

## 3. Methods

### 3.1. Synthesis of Chitosan Microspheres

Chitosan microspheres were prepared as per the method described by Dhawan et al. [17]. Briefly, a 1 : 1 ratio of light and heavy paraffin (125 mL) along with 0.5% of span 80 was taken as the continuous phase. The solution was stirred properly, and then 2.5% (w/v) of chitosan solution in acetic acid with 25 mg *α*-KG was added which constituted the dispersed phase. To the resulting oil in water emulsion 2.5 mL glutaraldehyde (25%) was then added dropwise at an interval of 15 min (15, 30, 45, and 60 min), and the resulting mixture was further stirred at 635 g for 2.5 h to obtain microspheres. The microspheres were separated by filtration and washed with n-hexane to remove adhered glutaraldehyde and paraffin. 

### 3.2. Synthesis of Gelatin Microspheres

Gelatin microspheres were prepared by the modification of the method reported by Mladenovskat et al. [18]. Briefly, a 10% gelatin solution (1 mL) containing 25 mg *α*-KG was added to the continuous phase containing 1 : 1 chloroform and toluene (5 mL) along with 0.25 g span 80. The mixture was stirred continuously at 158 g, and then 1.25 mL of 25% v/v glutaraldehyde was added dropwise at an interval of 15 min (15, 30, 45, and 60 min), and the resulting mixture was further stirred for 4 h to obtain microspheres. The resulting microspheres were washed by centrifugation at 2500 g for 5 min with 25% chloroform and 75% toluene followed by isopropanol and phosphate buffer saline (PBS).

### 3.3. *α*-KG Loading Efficiency of the Synthesized Microspheres

Gelatin and chitosan microspheres loaded with *α*-KG were crushed and dispersed in 1 mL sterile phosphate buffer saline (PBS), pH-7.4. The mixture was vortexed for 48 h at room temperature after which the tubes were centrifuged at 1000 g for 5 min. The supernatant was collected and further analyzed for *α*-KG to determine the encapsulation efficiency

(1) Encapsulation efficiency  =Calculated bioactive molecule concentrationTheoretical bioactive molecule concentration.

### 3.4. *In Vitro* Release Study of the Synthesized Microspheres

*In vitro* release test of *α*-KG from the synthesized microspheres was performed at 37°C. Loaded microspheres dispersed in PBS, pH-7.4, were kept in an Eppendorf tube on vortexer for 15 days. Every alternate day, the microspheres-containing tube was centrifuged, and the supernatant was collected and analyzed for *α*-KG concentration spectrophotometrically as reported earlier [19]. The cumulative release study was then used to calculate the amount of *α*-KG released over the period of 15 days. Fresh PBS was then added to each tube in order to obtain the release pattern of the respective microspheres. 

### 3.5. Synthesis of 3-D Cryogel Scaffold Incorporated with Microspheres

Pyrrole (0.13 M) was used for the synthesis of polypyrrole in the presence of iron chloride (FeCl_3_) and sodium dodecyl sulfate (SDS) as the oxidizing agent and surfactant, respectively. The resulting polypyrrole (PPy) solution was dialyzed for 48 h and further used for the cryogel synthesis. Chitosan (0.3%) and gelatin (1.6%) were dissolved in 2% acetic acid solution. To this was added the above dialysed PPy (5 mL) and mixed properly [20]. In order to synthesize cryogel incorporated microspheres, the filtered and dried chitosan and gelatin microspheres were weighed and added to the solution during the synthesis. This amount was calculated from the standard graph derived from the study of release kinetics of *α*-KG from the synthesized microspheres for 2 weeks. The solution was then poured in 5 mL syringe moulds and kept in cryostat at −12°C for 12 h. 

### 3.6. Cell Seeding on the Synthesized Scaffolds and Chitosan/Gelatin Microspheres

The monoliths (both incorporated with microspheres and nonincorporated with microspheres) were cut into 2 mm sections for seeding. The sections were sterilized by washing in gradient ethanol concentration (0%, 40%, 70%, and 100%) for a period of half an hour each time except in 70% ethanol in which the sections were left for 4-5 h for proper sterilization. The scaffolds were then washed 3 times with PBS containing 2% antibiotic for 15 min each. Thereafter the PBS was replaced with complete DMEM medium, and the scaffolds were allowed to completely saturate with media for 4 h. After this, the media were removed, and the scaffolds were seeded with the desired cell number.

Similarly for sterilization of microspheres, the calculated amount of *α*-KG containing microspheres for each well was dissolved in sterile tubes containing 1 mL PBS and 5% antibiotic for 1 h. After this the tubes were centrifuged and the supernatant was discarded and the microspheres were resuspended in complete DMEM medium. In the mean time neuro 2a cells were plated in 24 well plates and allowed to adhere for 6 h after which the above re-suspended chitosan and gelatin microspheres were added individually to the wells. For all the experimental setups the seeding density of 0.5 × 10^4^ was kept constant.

### 3.7. Delivery of *α*-KG to the Cells

*α*-KG (2 mM) was delivered in three ways to the cells. Firstly, it was administered exogenously wherein *α*-KG was dissolved in the media to get a final concentration of 2 mM, and 1 mL was added to each well (2-D and 3-D controls were wells without *α*-KG). Secondly, chitosan and gelatin microspheres incorporated with *α*-KG were administered to the 24-well plates with the cryogel scaffold (3-D) as well as without the scaffold (2-D) containing neuro 2a cells with 1 mL media. Thirdly, both gelatin and chitosan microspheres incorporated in the cryogel separately, seeded with neuro 2a cells were also filled with 1 mL media. For further biochemical analysis and experiments, the media were collected every third day till a period of 2 weeks without changing the media in the wells. 

### 3.8. Scanning Electron Microscopy (SEM) and Fluorescence Staining

The synthesized chitosan, gelatin microspheres, and the cryogels incorporated with and without the microspheres were analyzed by SEM. Further cell material interactions were also studied. The dried samples were gold coated for better imaging using a sputter coater (Vacuum Tech, Bangalore, India). The analysis was done at 20 kV and 3.5 mm spot size (FEI Quanta 200) in high vacuum conditions.

The cell seeded scaffolds were fixed with 2.5% glutaraldehyde for 4 h and then dried using gradient ethanol concentration (20%, 40%, 70%, and 100%) for 15 min each. The scaffolds were then cut into 100 *μ*m sections using Cryotome (Microm HM560, Thermo scientific) and further analyzed for the neuro 2a cells in the presence and absence of *α*-KG using fluoroscein diacetate stain (FDA). 

### 3.9. Cell Proliferation Assay

In order to analyze the cell proliferation in all the three cases, MTT was done. Every third day, the samples were collected in triplicates and assayed for their cell proliferation. The media were removed and 1 mL of MTT (0.05%) reagent was added to each well. After 4 h of incubation at 37°C, 1.5 mL of dimethyl sulfoxide (DMSO) was added to solubilize the formazon crystals to give purple colour which was then read spectrophotometrically at 570 nm. 

### 3.10. Estimation of Ammonia

Every third day, the media were collected in triplicates and assayed for ammonia by indophenol method [21]. In brief, 1 g of phenol and 5 mg sodium nitroprusside were dissolved in 100 mL deionized water to get solution I. Solution II was prepared by dissolving 2.158 g disodium hydrogen orthophosphate and 250 mg sodium hydroxide in 20 mL deionized water. Sodium hypochlorite solution (10 mL) was then added, and the final volume was made up to 50 mL with de-ionized water. For denaturing the protein, 10% sodium tungstate was used. For the analysis, 200 *μ*L spent media from the above three systems (exogenous, microspheres alone, and microspheres incorporated in cryogel—both chitosan and gelatin) were collected and mixed with 100 *μ*L 10% sodium tungstate and 100 *μ*L 1N H_2_SO_4_ in an eppendorf tube. The solution was properly mixed and then centrifuged at 1400 g for 10 min. Supernatant (100 *μ*L) was then collected and mixed with 500 *μ*L solution I and II. Samples were then incubated at 37°C for 35 min for the development of the colour. The absorbance of the resultant blue colour complex was measured at 625 nm. Blank had the same components as in the test sample except that de-ionized water was added instead of the spent media. The concentration of ammonia was calculated from straight line equation generated by plotting the standard graph. Estimation was done after every day for the duration of 15 days without changing the media. 

### 3.11. Estimation of Glucose

The samples collected for ammonia estimation were also analyzed for glucose in the media. In short, 250 *μ*L of the sample was mixed with 750 *μ*L of dinitrosalicylic acid (DNS) reagent. The solution was heated at 90°C for 30 min and was then allowed to cool. Thereafter, 10% sodium tungstate was added to the solution to stabilize the brownish-orange colour obtained. Absorbance was then taken at 540 nm. In blank, the media sample was replaced with de-ionized (DI) water.

### 3.12. Neurotransmitter Analysis

Media was collected in triplicates from the wells which were used for MTT assay to analyze the presence of neurotransmitters like glutamine, glutamate, and *γ*-amino butyric acid (GABA). HPLC (Waters, UV detector; 2414; Pump 515) with reverse phase hydrophobic C18 column (4.6 mm × 250 mm) was used for measurement of the samples according to the method reported by Donzanti and Yamamoto [22]. Briefly, the media samples collected were filtered through 0.2 *μ*m syringe filters. O-Phthalaldehyde/*β*-mercaptoethanol (OPA/*β*ME) reagent in the ratio 1 : 2 was mixed with 20 *μ*L filtered sample and the reaction was allowed for 2 min. After this, 20 *μ*L of the sample was injected in the C18 column maintained at 37°C. For analysis, 20% methanol and 3.5% acetonitrile were used as the mobile phase. 

## 4. Results and Discussion 

### 4.1. Synthesis of Chitosan/Gelatin Microspheres and Cryogel Scaffold

Chitosan and gelatin microspheres have long been used for the delivery of drug/bioactive molecules as they are biocompatible, degradable, and nontoxic in nature. These microparticles can result in controlled release of the incorporated bioactive molecule through diffusion initially and later because of degradation of these natural polymers by enzymes. Thus, molecules like *α*-KG, which undergo fast metabolism, can have a better shelf life and be available to the cells for longer duration when incorporated in microspheres. Moreover, the rational for choosing chitosan and gelatin for synthesis of the cryogel scaffold for growing neuro 2a cells is that chitosan is an important glycosaminoglycan which mimics the ECM of the native tissue. Moreover, the cationic nature of chitosan allows the negatively charged neural cells membrane to interact because of their electrostatic forces. Gelatin, apart from having RGD motifs for cell binding, provides elasticity to the synthesized scaffold as chitosan is very brittle in nature. Polypyrrole is a conducting polymer which is approved for use by FDA and has been reported to be extensively used for growing neural cells. The rational for the choice of the polymers for cryogel synthesis is already discussed in detail in our previous paper [19]. 

### 4.2. Microstructural Analysis by SEM

**Figure 1:**
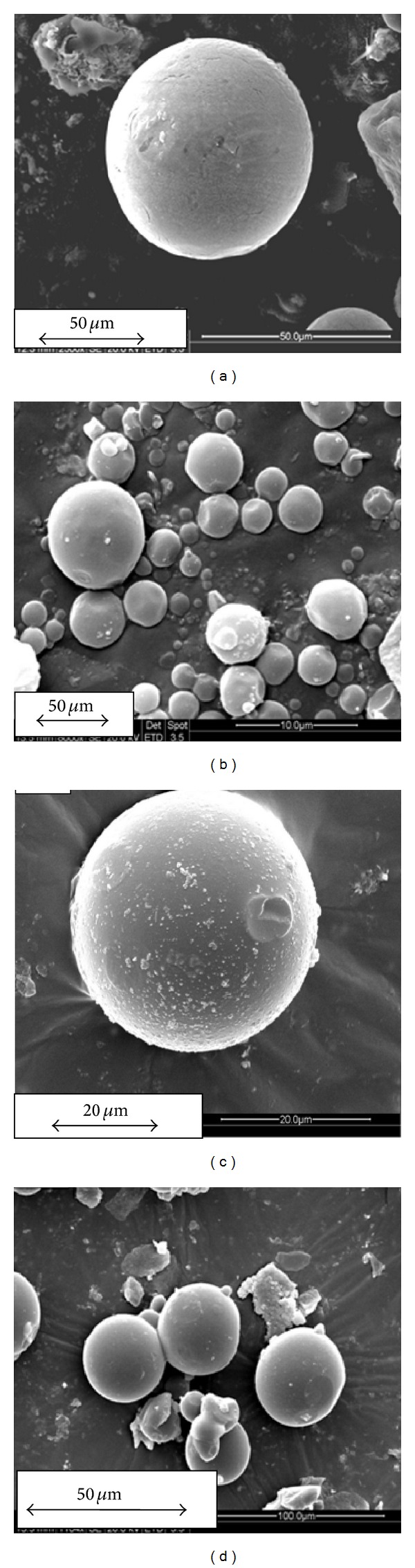
Scanning electron micrograph (SEM) of the synthesized microspheres: (a) magnified image of chitosan microsphere, (b) chitosan microspheres, (c) magnified image of gelatin microspheres, and (d) gelatin microspheres, (magnification: (a) 2500x; (b) 8000x; (c) 5000x; (d) 1160x).

**Figure 2:**
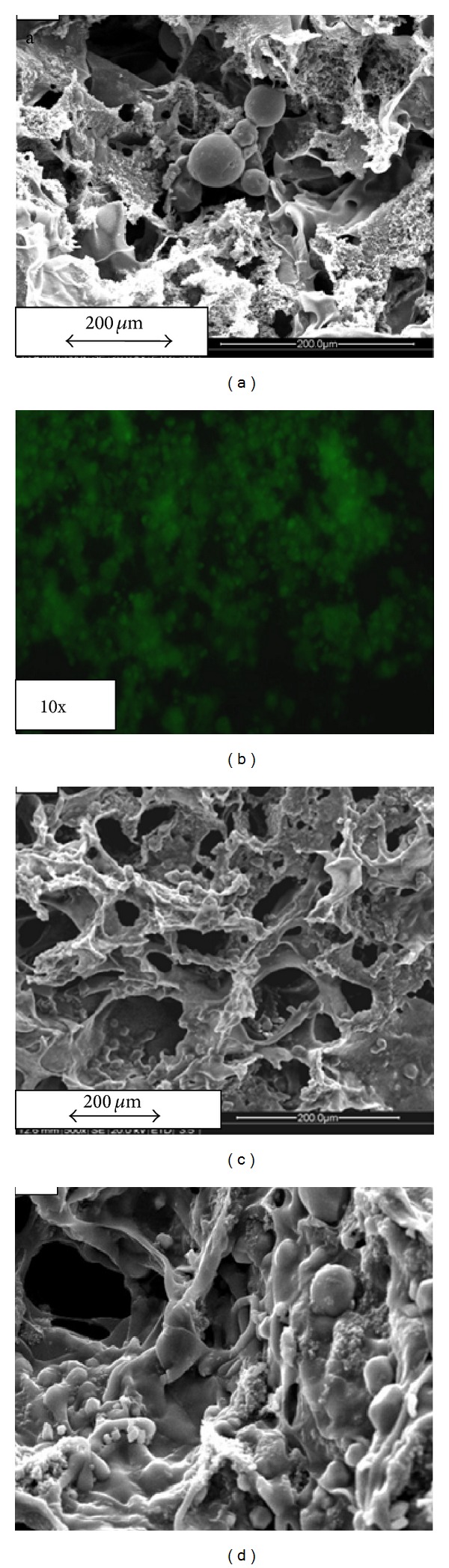
Scanning electron micrography (SEM) image of the following. (a) Microsphere (chitosan) incorporated cryogel (600x). (b) Fluoroscein diacetate (FDA) staining of neuro 2a on synthesized cryogel in the presence of added microspheres (gelatin). (c) SEM image of neuro 2a cells seeded in the presence of added chitosan microspheres (500x). (d) Magnified image of the image (c).

The synthesized microspheres were analyzed for their surface morphology by SEM. Figures 1(a) and 1(c) show the magnified image of chitosan and gelatin microspheres loaded with *α*-KG. The synthesized microspheres were mostly dispersed in nature and did not form much aggregates, which can be suitable as a delivery vehicle, for homogenous distribution of the loaded molecule (Figures 1(b) and 1(d)). Moreover, these synthesized chitosan and gelatin microspheres were mostly in the size range of around 25–30 *μ*m. Further, SEM was also performed in order to study the cryogels incorporated with the microspheres (Figure 2(a)). The image reveals the presence of gelatin microspheres in the synthesized porous cryogel scaffold along with the polypyrrole which is present throughout the cryogel walls. The microspheres added during the synthesis of cryogel can be observed to be embedded in the cryogel wall. FDA staining of neuro 2a cells seeded on the cryogel scaffold in the presence of *α*-KG showed homogenous cell distribution throughout the section (Figure 2(b)). Neuro 2a cells seeded on the synthesized cryogel scaffolds in the presence of exogenously added *α*-KG showed extension of their neurites in the SEM image apart from their uniform distribution (Figures 2(c) and 2(d)). 

### 4.3. Encapsulation Efficiency and *In Vitro *Release Study

**Figure 3:**
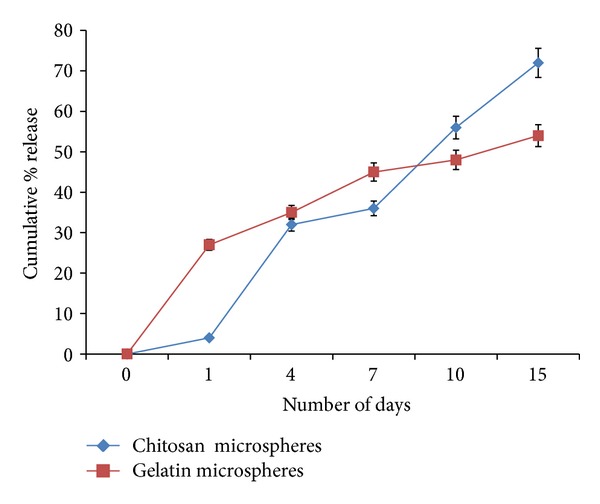
*In vitro *release pattern of synthesized chitosan and gelatin microspheres. Cumulative percentage release of the microspheres was analyzed for 15 days.

Encapsulation efficiency can be defined as the capacity of the microspheres to incorporate the drug/bioactive molecule. Synthesized chitosan and gelatin microspheres have been reported to have 76% and 80% encapsulation efficiency, respectively. These *α*-KG loaded microspheres were also studied for their release pattern in order to understand and control the duration and the amount of bioactive molecules to be added. The release pattern of *α*-KG depends on the loading efficiency, concentration of chitosan/gelatin, and the crosslinker concentration. There is a burst release in the initial 24 h and 48 h in the case of gelatin and chitosan microspheres, later to which an increasing trend was observed leading to 53% and 72% cumulative percentage release, respectively (Figure 3).

The amount of chitosan and gelatin microspheres to be added to the medium or incorporated in the synthesized cryogel was calculated from the standard graph and the release profile of *α*-KG incorporated microspheres. From this calculation, approximately 0.5 mg of chitosan and gelatin microspheres need to be added to get 2 mM concentration of *α*-KG. We have previously optimized 2 mM concentration of *α*-KG for mammalian cells used either for therapeutic purpose like monoclonal antibody production [23] or tissue engineering of fibroblasts and primary chondrocytes (isolated from goat knee joint) [24]. Therefore, in this study also, we continued to work with the same concentration and tried to analyze its effect on neural cell line, neuro 2a.

### 4.4. Cell Proliferation Assay

**Figure 4:**
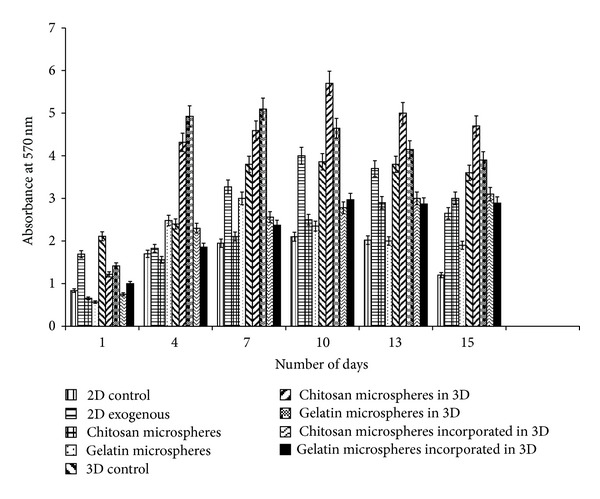
Cellular metabolic activity/proliferation in the presence and absence of alpha ketoglutarate (*α*-KG) was analyzed by MTT assay. Neuro 2a metabolic activity/proliferation on 2-D and 3-D control (*α*-KG absent) as well as *α*-KG containing samples wherein *α*-KG was delivered in the following three different ways: (i) exogenously in free form in 2-D, (ii) chitosan or gelatin microspheres containing *α*-KG in both 2-D and 3-D system, and (iii) *α*-KG containing chitosan or gelatin microspheres incorporated in the synthesized chitosan-gelatin-polypyrrole cryogel were analyzed. All the experiments were performed in triplicates, and the Student's-*t* test was performed to obtain the *P* value. *P* < 0.05.

MTT assay was performed to study the cellular proliferation of neuro 2a in the presence of *α*-KG, when administered by three different modes as described previously in the methods section. The results showed an increased proliferation in the presence of *α*-KG both in 2-D as well as 3-D when compared with their respective controls (without *α*-KG) (Figure 4). It had also been reported previously that cell proliferation of intestinal cells increased when they were administered with *α*-KG [25]. Similarly, it has been shown that mesangial cells when supplied with extracellular ATP significantly increased the cell count by 35.1% [26]. In this study, when *α*-KG was administered exogenously in the free form, it was readily taken up by the cells. *α*-KG being a potent TCA cycle intermediate is metabolized to release CO_2_ and H_2_O as the ultimate product along with ATP, the energy currency of the cells. This ATP is used up by the cells for various biological and biochemical reactions. The 2-D samples displayed a bell curve for MTT assay as they became confluent earlier due to lesser surface area of tissue culture plates in which they were seeded, and thus, their MTT values decreased after 10 days as compared to the 3-D cultures. The synthesized scaffolds because of their architecture and porous nature had a higher surface area leading to higher proliferation for a longer period of time. The cell line used (neuro 2a) has a short doubling time of <8 h [27], and so, free exogenous delivery seems to have an important role, but for cell lines where the cell growth is slow as in case of chondrocytes [28], sustained and prolonged delivery of *α*-KG is required. Therefore, delivery via microspheres seems to be a better approach. The encapsulation of the bioactive molecule prevents the loss and degradation of the molecule apart from providing controlled release. The wells loaded with microparticles showed slightly slow proliferation in the first 24 h as compared to the control which can be attributed to the fact that the cells took some time to adjust to the foreign particles present in the well. In the later time points, the microparticles being compatible in nature allowed the cells to multiply, and their proliferation increased compared to their control. Gelatin microspheres due to their faster degradation rate as compared to the chitosan microspheres led to an increased release of *α*-KG in the media. This resulted in enhanced proliferation of the neuro-2a cells for the first week after which their proliferation decreased as compared to the chitosan microspheres. The slow degrading chitosan micro-particles allowed the release of *α*-KG through diffusion and osmosis which continued for longer duration of time. Similar results were obtained in the 3-D system for gelatin and chitosan microspheres though their proliferation was significantly higher when compared to the 2-D system and their control. We can conclude that micro-particles can be selected for delivery as per the cell line and duration of delivery. Gelatin micro-particles can be preferred over the chitosan micro-particles when used for delivery of drug/bioactive molecules to the fast growing cells like carcinoma cells, fibroblasts, and so forth. In this study, we also analyzed the third delivery system wherein the cryogels were incorporated with synthesized microparticles with the concentration of 2 mM. The results showed that there was a continuous increase of cell proliferation for two weeks both in chitosan and gelatin incorporated cryogels. When compared to the other 3-D systems, the cell proliferation of these systems was less which could be due to several reasons. We speculate that the incorporation of micro-particles and polypyrrole in the cryogel during the synthesis procedure blocked the pores leading to inhibition in the convective flow of nutrients and waste. Moreover, it also prevented the cells adherence on the pore walls due to which the cell number and so the cell proliferation reduced.

### 4.5. Estimation of Ammonia

**Figure 5:**
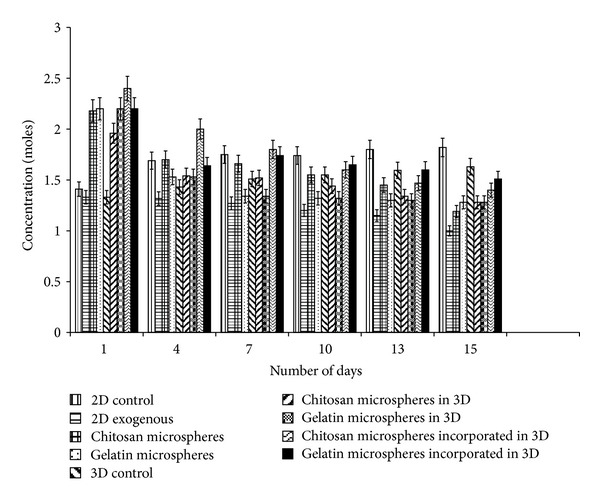
Ammonia measurements in 2-D and 3-D control as well as *α*-KG containing samples were done by indophenols method. Delivery of *α*-KG was done in three different ways. (i) Exogenously in free form in 2-D. (ii) Chitosan or gelatin microspheres containing *α*-KG in both 2-D and 3-D system. (iii) *α*-KG containing chitosan or gelatin microspheres incorporated in the synthesized chitosan-gelatin-polypyrrole cryogel. All the experiments were performed in triplicates, and the Student's-*t* test was performed to obtain the *P* value. *P* < 0.05.

Batch cultures lead to ammonia accumulation as the cells during their proliferation consume glucose, glutamine as their source of energy and in return release ammonia. Ammonia is toxic to the cells leading to inhibition of various cellular activities like protein glycosylation, protein synthesis, cell viability, and so forth. Unlike the *in vivo* conditions in which ammonia is metabolized to urea, in the *in vitro* conditions it remains either as ammonia or ammonium ion leading to cell toxicity. Moreover, L-glutamine, present in the media, is unstable in nature and degrades spontaneously into ammonia thus building up ammonium toxicity in the media during long culture conditions. Therefore, in order to regulate this toxicity, we added *α*-KG in the media by various means and observed significant difference in ammonia accumulation in the media over the culture period of two weeks. In both 2-D and 3-D control, the ammonia concentration increased over the period of time. In comparison, all the systems with *α*-KG showed a decline in ammonia accumulation wherein exogenous supply of *α*-KG showed a continuous and significant decrement of ammonia accumulation compared to the others. Although chitosan and gelatin microspheres decreased the ammonia concentration to the same level in the media at the end of the two weeks, this decline started later as compared to the system where *α*-KG was supplied exogenously. This was because *α*-KG from the microspheres is released mainly through diffusion and osmosis as compared to the freely available *α*-KG in the above case. Gelatin microspheres due to their faster degradation rate also helped in faster reduction of ammonium accumulation compared to the chitosan microspheres. Similar results were also obtained for 3-D cultures with added gelatin and chitosan microspheres. We speculate that the synthesized cryogel scaffolds also in some way allowed less buildup of ammonia concentration in the first 24 h which needs to be further explored. It may be that the easier degradation of these microspheres by the cells (due to direct interaction of the cells and microspheres) might have contributed to the sudden rise in ammonia accumulation in 2-D system. Chitosan and gelatin both having amine groups might have led to this accumulation during their degradation. Although cryogels incorporated with the microspheres showed lesser reduction in ammonia accumulation, the decline was significantly more when compared with the control. A declining trend was observed for both the microspheres incorporated cryogel as shown in Figure 5. 

### 4.6. Estimation of Glucose

**Figure 6:**
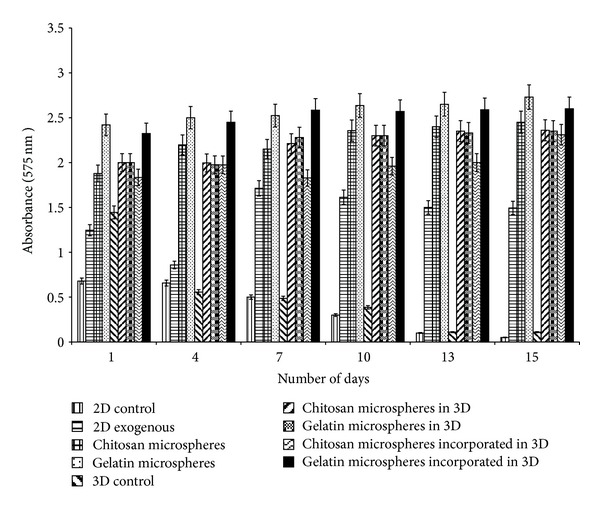
Glucose concentration in the spent media was analyzed by Dinitrosalicylic acid (DNS) method both in control (2-D and 3-D) as well as *α*-KG containing samples wherein *α*-KG was delivered in the following three different ways: (i) exogenously in free form in 2-D, (ii) chitosan or gelatin microspheres containing *α*-KG in both 2-D and 3-D system, and (iii) *α*-KG containing chitosan or gelatin microspheres incorporated in the synthesized chitosan-gelatin-polypyrrole cryogel. All the experiments were performed in triplicates, and the Student's-*t* test was performed to obtain the *P* value. *P* < 0.05.

Glucose is essential for the cells to undergo aerobic pathway and is well established by Korinkova and Lodin with their experiments [29]. Cells use the energy released by glucose metabolism for their growth and multiplication thus leading to cellular proliferation. The graph (Figure 6) indirectly shows the consumption of glucose by the cells for their proliferation and other metabolic activities by assaying the glucose concentration left in the media. The media used had 4500 mg/L glucose as the initial concentration. During the experiment, the glucose level of both 2-D and 3-D control decreased over the period reaching a minimum at the end of the 15 days. To the contrary the systems with either exogenous *α*-KG, microspheres containing *α*-KG, or microspheres incorporated cryogel showed decreased but almost constant glucose consumption during the culture period of 15 days. It has been already reported that few mammalian cell lines start using glutamine, when supplied exogenously, as an alternative source of energy for rapidly dividing cells, and in return there is a decrement in glucose consumption [30]. Wherever *α*-KG was supplied to the cells, by any means, the glucose consumption decreased and the oxidation of glutamine increased. This might be because *α*-KG in the presence of toxic ammonia is converted to glutamine and glutamate wherein this synthesized glutamine is used as an alternative source of energy for the cells. In the systems where *α*-KG was supplied either (i) exogenously, (ii) through microspheres, or (iii) incorporated in the cryogels, the glucose concentration present in the spent media became almost constant by the end of the first week. Thereby, it can be said that there was reduction in the glucose consumption in the presence of *α*-KG as compared to the control wherein there was a continuous decrease in the glucose concentration over the period of two weeks. 

### 4.7. Neurotransmitter Analysis

**Figure 7:**
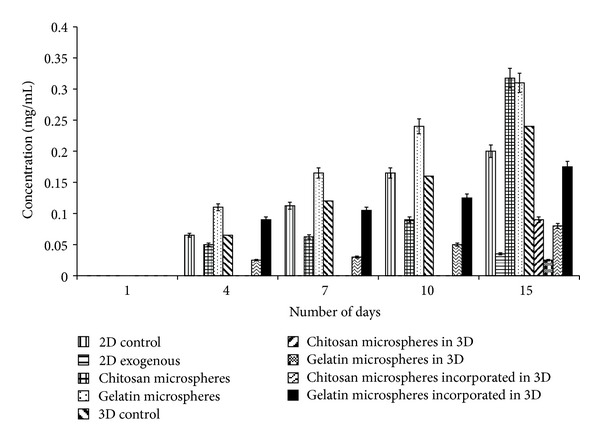
Estimation of glutamate concentration by HPLC was done. It was analyzed both in control (2-D and 3-D) as well as *α*-KG containing samples wherein *α*-KG was delivered in the following three different ways: (i) exogenously in free form in 2-D, (ii) chitosan or gelatin microspheres containing *α*-KG in both 2-D and 3-D system, and (iii) *α*-KG containing chitosan or gelatin microspheres incorporated in the synthesized chitosan-gelatin-polypyrrole cryogel. All the experiments were performed in triplicates, and the Student's-*t* test was performed to obtain the *P* value. *P* < 0.05.

**Figure 8:**
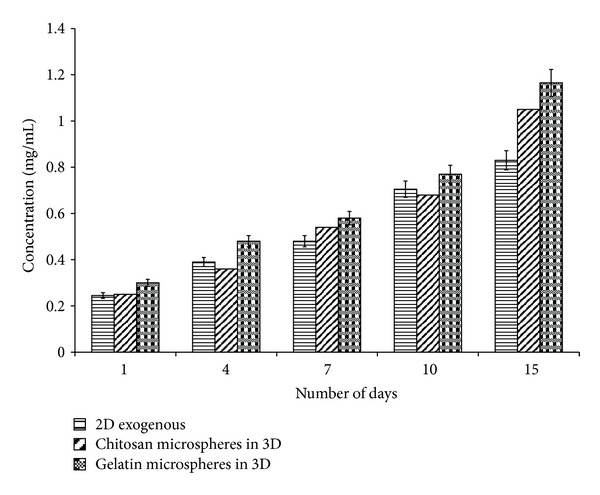
Estimation of glutamine concentration by HPLC was done. It was analyzed both in control (2-D and 3-D) as well as *α*-KG containing samples wherein *α*-KG was delivered in the following three different ways: (i) exogenously in free form in 2-D, (ii) chitosan or gelatin microspheres containing *α*-KG in both 2-D and 3-D system, and (iii) *α*-KG containing chitosan or gelatin microspheres incorporated in the synthesized chitosan-gelatin-polypyrrole cryogel. All the experiments were performed in triplicates, and the Student's-*t* test was performed to obtain the *P* value. *P* < 0.05.

The analysis of glutamate, glutamine was done using HPLC primarily because *α*-KG converts to glutamate after reacting with ammonium ion in the presence of glutamate dehydrogenase and NADPH + H^+^. Further, this glutamate converts to glutamine when ATP and NH_4_^+^ are present along with the enzyme glutamate synthetase [31]. Glutamate and glutamine are important amino acids and are involved in protein synthesis. Apart from this, glutamate also functions as a neurotransmitter. An increasing trend in glutamate concentration was observed in all the systems (Figures 7 and 8) except the 2-D system in which exogenously *α*-KG was supplied and the 3-D system in which chitosan and gelatin microspheres were added in the media. In these systems, glutamate was observed in the media in 15 days whereas glutamine was observed throughout the experiment. Glutamate eventually converts to glutamine and then finally to *α*-KG; therefore, we consider that this conversion is occurring faster in these systems, and this theory also correlates with their proliferation. As can be seen through MTT assay, the cell proliferation of these three systems is faster as compared to the other systems, and we speculate that the cells require more energy for their proliferation, and, thus there is faster conversion of glutamate to glutamine leading to its constant increase during the experiment. It is known that glutamate decarboxylation in the presence of the enzyme glutamate decarboxylase leads to the synthesis of GABA [32] which serves as a neurotransmitter, but in our case instead of this glutamate—GABA cycle glutamate—glutamine cycle [33] is predominant, and thus, we could not find GABA in our experiments. The glutamine concentration present in the DMEM media initially is 4 mM (584.6 *μ*g/mL), but it is well known that this is chemically unstable [34] and degrades within 48 h, and so that we consider that the glutamine content analyzed during HPLC analysis is mainly the one obtained through *α*-KG conversion. 

## 5. Conclusion

In conclusion, there is a varying degree of delivery of the bioactive molecule when different modes are used. The selection would depend on their application and ultimate use. Mode of delivery is governed by various factors primarily being the duration of exposure and the cell line to which the molecule is to be delivered. In order to achieve optimum results in tissue engineering, there is a requirement for a particular cell seeding density which in most cases is difficult to attain. Therefore, *α*-KG can be used as an important delivery metabolite in order to overcome this inhibition without any negative effects on cells. In this work, it is clearly observed that free exogenous supply of *α*-KG to the cells showed better results compared to the delivery by microspheres as well as by microspheres incorporated cryogel. Moreover, the degradation rate of these microspheres did alter the delivery of *α*-KG and thus the cellular proliferation and other biochemical analysis. Therefore, we can conclude that delivery via free form is the most preferred for short duration, whereas delivery by microspheres can be selected for longer duration. Comparatively, microspheres incorporated cryogels showed the least effect due to the above mentioned reasons.
